# High-Fat, High-Cholesterol Diet Influence on Matrix Metalloproteinases and Transcription of c-Jun and TGF-βin Cardiac Muscle of ApoE (-/-) Mice

**DOI:** 10.3390/ijms27093888

**Published:** 2026-04-27

**Authors:** Michał Kowara, Katarzyna Czarzasta, Michał Jędrzejewski, Łukasz Koperski, Agnieszka Segiet-Święcicka, Robert Wrzesień, Marek Kuch, Agnieszka Cudnoch-Jędrzejewska

**Affiliations:** 1 Department of Experimental and Clinical Physiology, Laboratory of Centre for Preclinical Research, Medical University of Warsaw, Banacha 1b, 02-097 Warsaw, Poland; 2 Krajmed Medical Center, Bacha 2, 02-743 Warsaw, Poland; 3 Department of Pathology, Medical University of Warsaw, Pawińskiego 7, 02-106 Warsaw, Poland; 4 Central Laboratory of Experimental Animals, Laboratory of Centre for Preclinical Research, Medical University of Warsaw, Banacha 1b, 02-097 Warsaw, Poland; 5 Chair and Department of Cardiology, Hypertension and Internal Medicine, Medical University of Warsaw, Kondratowicza 8, 03-242 Warsaw, Poland

**Keywords:** ApoE (-/-) mice, atherosclerosis, matrix metalloproteinases, high-fat, high-cholesterol diet, mRNA

## Abstract

A high-fat, high-cholesterol diet (HFHCD) has a lipotoxic effect on the heart. It not only leads to the development of atherosclerosis but also influences the extracellular matrix within the heart. The aim of the study was to investigate the effect of HFHCD on matrix metalloproteinases MMP-2, MMP-9, MMP-13, and MMP-14 expression in both the cardiac tissue and plasma of ApoE (-/-) mice and on mRNA expression of c-Jun and TGF-β in the cardiac tissue of both ApoE (-/-) mice and wild-type C57BL/6J mice. The study was carried out on two groups of ApoE (-/-) mice: (1) mice from 10 weeks of age that were kept on a HFHCD (*n* = 10) for the following 14 weeks; (2) control mice (NFD, *n* = 10) that were kept on a standard, normal-fat diet for the same time as the HFHCD. Additionally, 10 wild-type (WT) mice on a standard, normal-fat diet were also included in the study for mRNA analysis of c-Jun and TGF-β. Atherosclerotic plaque, intima, and media dimensions were assessed in the aortas of the ApoE (-/-) mice by histopathology. Concentrations of MMP-2, MMP-9, MMP-13, and MMP-14 were assessed by ELISA both in cardiac tissue and in the plasma of the ApoE (-/-) HFHCD and ApoE (-/-) NFD mice, while the mRNA expression of c-Jun and TGF-β was assessed by RT-PCR in both the ApoE (-/-) and WT groups. The results demonstrate a significantly increased MMP-9 concentration in the cardiac tissue of the HFHCD mice compared to the NFD mice (2.83 ng/mL vs. 1.91 ng/mL, *p* = 0.006), and a moderate correlation between the cardiac and plasmatic MMP-9 in ApoE (-/-) mice (r = 0.492, *p* = 0.0398). Moreover, although the mRNA expression of c-Jun and TGF-β did not differ between NFD and HFHCD ApoE (-/-) mice, the c-Jun expression was significantly elevated in the WT group compared with both ApoE (-/-) groups. The study demonstrated that a high-fat, high-cholesterol diet increases MMP-9 concentration in cardiac tissue, which might reflect its influence on the extracellular matrix within the heart.

## 1. Introduction

A high-fat, high-cholesterol diet influences metabolism in human and mammalian organisms and leads to hyperlipidemia, one of the most important factors causing atherosclerosis-related disease, especially coronary artery disease [1,2]. The association between a high-fat, high-cholesterol diet and an increased atherosclerotic burden in the aorta has been well established in animal models of atherosclerosis, especially ApoE (-/-) mice [3,4]. It was also demonstrated that a high-fat and high-cholesterol diet induces more subtle changes that are considered pre-atherogenic, such as an increased intima–media ratio [5]. However, hyperlipidemia not only affects the arteries but also directly damages cardiac tissue by modifying the extracellular matrix (ECM), thereby inducing cardiac fibrosis [6]. The ECM is a dynamic structure that undergoes constant physiological remodeling, during which existing proteins are degraded by matrix metalloproteinases (MMPs) and replaced by newly synthesized proteins [7,8]. The MMPs that are crucial in extracellular matrix remodeling are collagenases MMP1 and MMP13, gelatinases MMP2 and MMP9, as well as membrane-type matrix metalloproteinase MMP-14 [9]. The MMPs are under epigenetic control of transcription factor c-Jun, which binds to the MMPs’ promoters and stimulates their transcription [10,11,12]. The c-Jun activates a pro-fibrotic response in cardiomyocytes and influences ECM turnover within the cardiac tissue. Its expression, assessed by the mRNA level, is regulated in response to external stimuli [13,14]. Another factor that regulates ECM protein expression and maintains a balance between matrix metalloproteinases and their inhibitors is the cytokine TGF-β [15,16]. This study aimed to investigate differences in the concentrations of MMP-2, MMP-9, MMP-13, and MMP-14 in the cardiac tissue and plasma of Apo (-/-) mice fed a high-fat, high-cholesterol diet (HFHCD) compared with ApoE (-/-) mice fed a normal-fat diet (NFD). Although it was revealed that a high-fat, high-cholesterol diet affects MMPs expression in ApoE (-/-) mice, these studies investigated MMPs by immunohistochemical methods within the vessels, not in the heart or in correlation with their plasma levels [17,18]. In addition, our study not only compared the mRNA expression levels of c-Jun, and TGF-β in both groups of ApoE (-/-) mice (HFHCD and NFD) but also in a group of (WT) mice, i.e., C57BL/6 mice without an *ApoE* mutation. Although it is known that a high-fat, high-cholesterol diet induces alterations in gene expression in aortic tissue and in cardiac TGF-β signaling in ApoE (-/-) mice, its direct influence on c-Jun and TGF-β mRNA levels in cardiac tissue remains underexplored [19,20,21]. ApoE (-/-) mice were selected because this model is not only optimal for studying atherosclerotic plaque development but also for studying the processes in which extracellular matrix remodeling is crucial (e.g., aortic aneurysm formation) [22,23]. The LDL to HDL ratio in ApoE (-/-) mice is similar to the HDL/LDL ratio in humans, and because of the similarity in cholesterol transport and metabolism, atherosclerotic lesions in ApoE (-/-) mice resemble atherosclerotic lesions in humans in their sites of predilection and progression stages [24]. It was also demonstrated that a high-fat, high-cholesterol diet induces alterations in extracellular matrix proteins (especially collagens) in C57BL/6 mice; therefore, ApoE (-/-) and C57BL/6 mice (WT—wild-type) were selected for our study [25].

## 2. Results

### 2.1. Characteristics of the Animals

**Figure 1:**
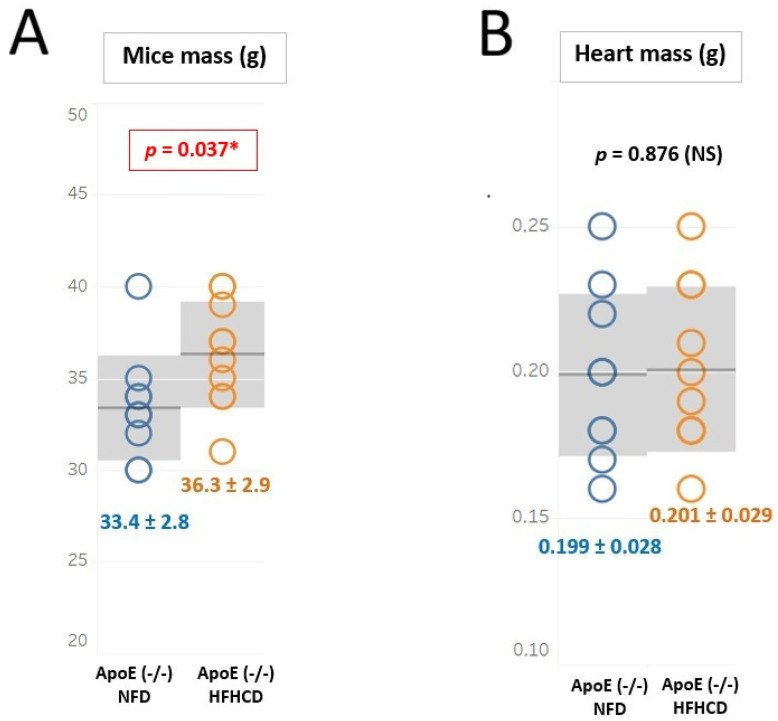
Mice mass and heart mass distribution in ApoE (-/-) mice fed with a non-fat diet (NFD), blue color compared with ApoE (-/-) mice fed with a high-fat, high-cholesterol diet (HFHCD), orange color expressed as mean ± SD (gray area) (**A**,**B**) * *p* < 0.05, statistically significant value. The normal distribution and equality of variances were confirmed by the Shapiro–Wilk test and the Levene test, respectively. Student’s *t*-test was applied. NS—non significant.

The statistical analysis showed that the body weight was significantly different between the examined groups of mice (HFHCD mice, 36.5 [34.2, 38.5] g vs. NFD mice, 33.0 [32.2, 34.0] g vs. WT mice, 33.0 [32.0, 34.0] g; *p* < 0.01). On the other hand, the heart weight per 100 g body weight did not differ significantly between the examined groups of mice (HFHCD mice, 0.561 ± 0.112 g/100 g; body weight vs. NFD mice, 0.598 ± 0.078 g/100 g; body weight vs. WT mice, 0.580 ± 0.068 g/100 g; body weight, *p* = NS). The distribution of body weight and heart weight in HFHCD and NFD mice is shown in Figure 1.

### 2.2. Results of Histopathological Analysis of Atherosclerotic Plaque

**Figure 2:**
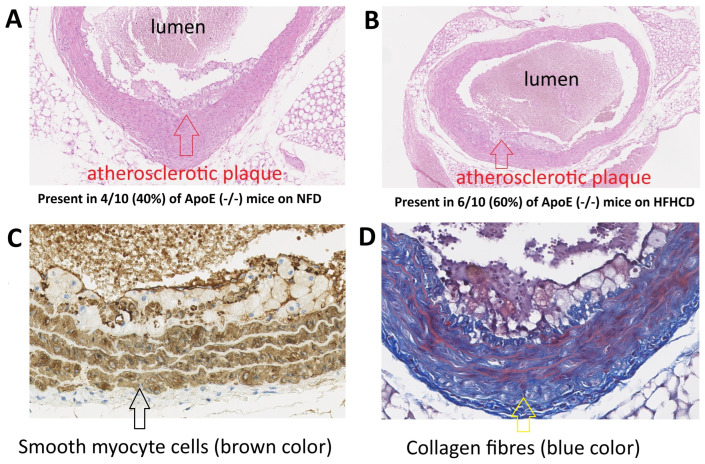
Atherosclerotic plaque in ApoE (-/-) mice shown by hematoxylin and eosin (H&E) staining: (**A**) on a standard, normal-fat diet (NFD) (10× magnification) and (**B**) on a high-fat, high-cholesterol diet (HFHCD) (10× magnification). (**C**) Atherosclerotic plaque staining for myocytes (immunohistochemical staining for SMA) and (**D**) atherosclerotic plaque staining for collagen fibers (Masson’s trichrome staining).

Histopathological analysis revealed the presence of a well-developed atherosclerotic plaque with a superficial thin fibrous cap and focal loss of endothelium in both NFD mice and HFHCD mice (four mice with developed atherosclerotic plaque confirmed by the histological study in the NFD group and six mice with developed atherosclerotic plaque confirmed by the histological study in the HFHCD group, χ^2^ test *p* = 0.37) (Figure 2). Additional staining in both groups revealed details of the structure of individual atherosclerotic plaques, including, for example, the presence of myocytes in their composition and the continuity of the fibrous cap.

**Figure 3:**
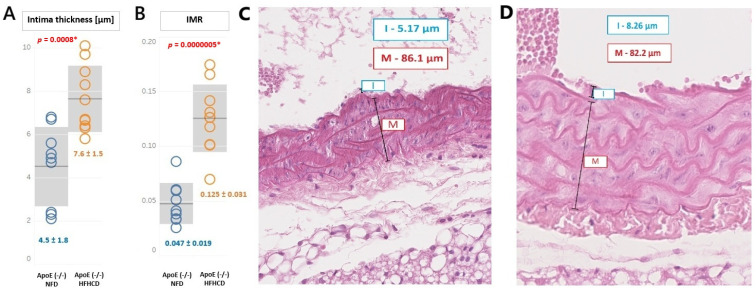
(**A**) Intima thickness (mean ± SD, gray area) and (**B**) intima–media radio (IMR) (mean ± SD, gray area) in the ApoE (-/-) mice on a high-fat, high-cholesterol diet (orange color) compared to a standard, normal-fat diet (blue color). * *p* < 0.05, statistically significant value. The normal distribution and equality of variances were confirmed by the Shapiro–Wilk test and Levene test, respectively. Student’s *t*-test was applied. (**C**) Exemplary intima (I), media (M), and IMR calculation in a mouse from the NFD group and (**D**) exemplary intima (I), media (M), and IMR calculation in a mouse from the HFHCD group.

However, the HFHCD induced a significant endothelial modification. The intima dimension in the ApoE (-/-) HFHCD was significantly higher than in the ApoE (-/-) NFD mice (7.6 ± 1.5 μm vs. 4.5 ± 1.8 μm, *p* = 0.000802). Similarly, the intima–media ratio (IMR) was also significantly higher in the ApoE (-/-) HFHCD mice (0.125 ± 0.031 vs. 0.047 ± 0.019, *p* = 0.0000005). These results are presented in Figure 3.

### 2.3. Levels of MMP in the Myocardium

**Figure 4:**
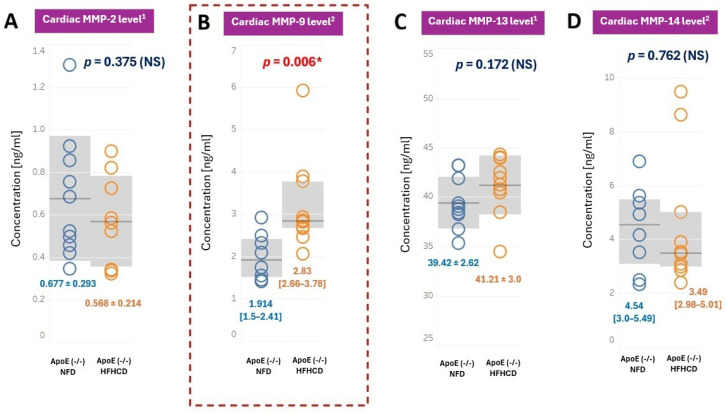
Cardiac concentrations of (**A**) MMP-2, (**B**) MMP-9, (**C**) MMP-13, and (**D**) MMP-14 in the ApoE (-/-) mice on NFD (blue color) compared to ApoE (-/-) mice on HFHCD (orange color). ^1^ normal distribution, concentrations expressed as mean ± standard deviation (SD) (gray area), and homogenous variances. Student’s *t*-test was applied for comparison. ^2^ normality rejected and concentrations expressed as median [interquartile range (IQR)] (gray area). The Mann–Whitney U-test was applied. NS—non-significant; * *p* < 0.05 is considered statistically significant.

ELISA analyses revealed that MMP-9 levels in the myocardium were significantly higher in the HFHCD mice compared with the NFD mice (*p* = 0.006) (Figure 4B). In contrast, the levels of MMP-2, MMP-13, and MMP-14 in the myocardium did not differ significantly between both groups of ApoE (-/-) mice (Figure 4A,C,D).

### 2.4. Levels of MMPs in Plasma and Correlations Between Myocardium and Plasma Levels

**Figure 5:**
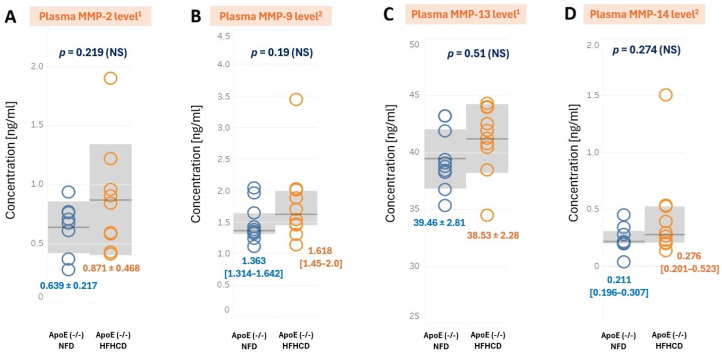
Plasma concentrations of (**A**) MMP-2, (**B**) MMP-9, (**C**) MMP-13, and (**D**) MMP-14 in the ApoE (-/-) mice on NFD (blue color) compared to ApoE (-/-) mice on HFHCD (orange color). ^1^ normal distribution, concentrations expressed as mean ± standard deviation (SD) (gray area), and homogenous variances. Student’s *t*-test was applied for comparison. ^2^ normality rejected and concentrations expressed as median [interquartile range (IQR)] (gray area). The Mann–Whitney U-test was applied. NS—non-significant; *p* < 0.05 considered statistically significant.

ELISA analyses revealed no significant differences in plasma concentrations of MMP-2, MMP-13, MMP-9 and MMP-14 between ApoE (-/-) NFD and ApoE (-/-) HFHCD (Figure 5).

**Figure 6:**
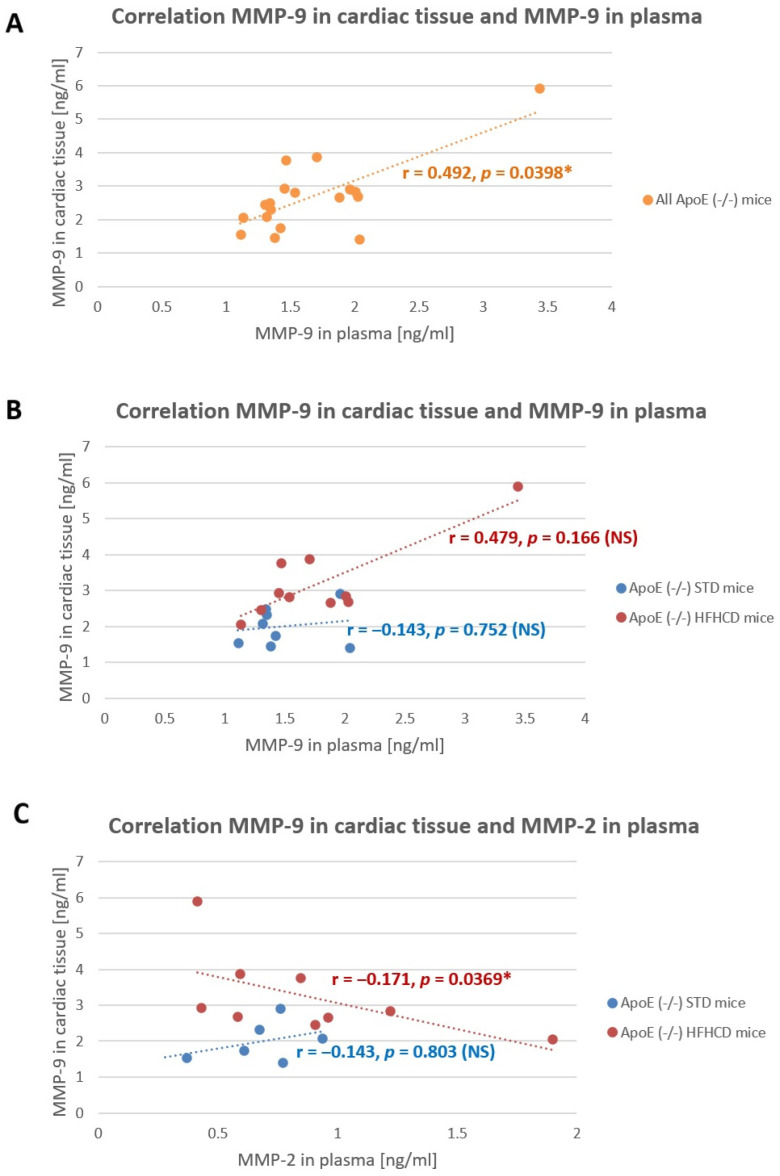
Correlations between (**A**) MMP-9 in cardiac tissue and MMP-9 in plasma in the entire population of ApoE (-/-), (**B**) MMP-9 in cardiac tissue and MMP-9 in plasma separately for the ApoE (-/-) NFD mice and the ApoE (-/-) HFHCD mice, (**C**) MMP-9 in cardiac tissue and MMP-2 in plasma separately for the ApoE (-/-) NFD mice and the ApoE (-/-) HFHCD mice. Correlation analysis in (**A**–**C**) by the Spearman ρ coefficient due to the lack of normal distribution in MMP-9 cardiac tissue and in plasma. Dash color lines (orange, red, blue) stands for trend lines. * *p* < 0.05 considered statistically significant. NS—non significant.

Nevertheless, a significant positive correlation was found between plasma MMP-9 and cardiac tissue MMP-9 when all ApoE (-/-) were analyzed together (r = 0.492, *p* = 0.0398). When the correlations in ApoE (-/-) mice were analyzed separately for the NFD and the HFHCD, there was still a positive correlation between MMP-9 in plasma and in cardiac tissue in the HFHCD mice, but it did not reach statistical significance (r = 0.479, *p* = 0.166). Moreover, a slight but significant negative correlation was found between the MMP-2 concentration in plasma and the MMP-9 concentration in cardiac tissue (r = −0.171, *p* = 0.0369) (Figure 6). No other significant, reliable correlations were found between the other MMPs in the cardiac tissue and in the plasma.

### 2.5. Expression of c-Jun and TGF-β in Plasma

**Figure 7:**
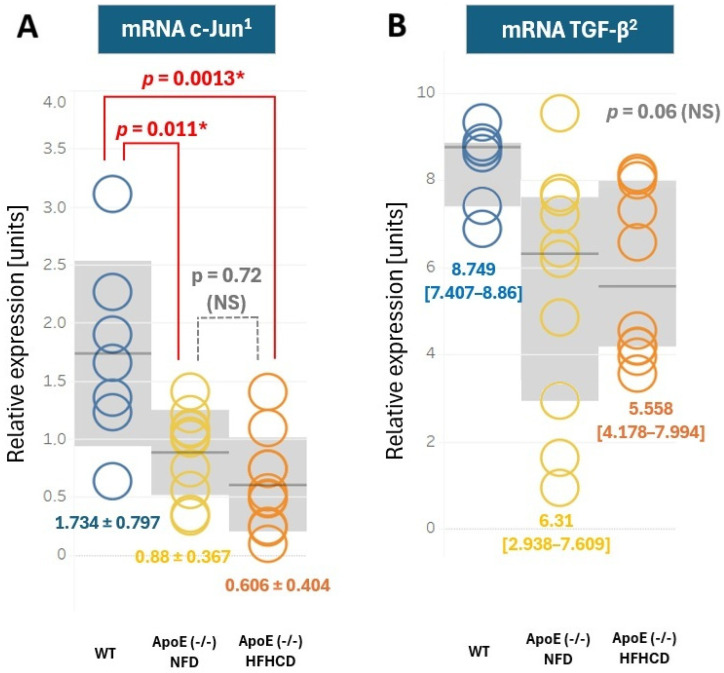
(**A**) c-Jun expression in the C57BL/6J mice (WT); the ApoE (-/-) mice on a standard, normal-fat diet (NFD); and the ApoE (-/-) mice on a high-fat, high-cholesterol diet (HFHCD). The relative expression is expressed as mean ± SD (gray area). (**B**). TGF-β expression in the C57BL/6J mice (WT); the ApoE (-/-) mice on a standard, normal-fat diet (NFD); and the ApoE (-/-) mice on a high-fat, high-cholesterol diet (HFHCD). The relative expression is expressed as median [IQR] (gray area). WT mice—blue color, ApoE (-/-) mice on NFD—yellow color, ApoE (-/-) mice on HFHCD—orange color. ^1^ normal distribution in the Shapiro–Wilk test, homogenous variances, and F-ANOVA test with Scheffe post hoc analysis. ^2^ normal distribution in the Shapiro–Wilk test; the homogeneity of variances was rejected in the Levene test. The ANOVA Kruskal–Wallis test was applied. * *p* < 0.05 and is considered statistically significant. NS—non significant.

The RT-PCR analysis of cardiac tissue revealed no significant differences in c-Jun and TGF-β expression when ApoE (-/-) HFHCD were compared with an ApoE (-/-) NFD. However, when the wild-type C57BL/6J mice were compared with both the NFD and HFHCD mice, significant differences were found in c-Jun expression (between the WT and NFD mice, 1.734 ± 0.797 vs. 0.880 ± 0.367, *p* = 0.0011 and between the WT and HFHCD mice, 1.734 ± 0.797 vs. 0.606 ± 0.404, *p* = 0.0013). Moreover, a trend was also observed in the mRNA TGF-β expression, which was higher in the WT mice (median 8.749, interquartile range [7.407–8.86]) compared to the NFD (6.31 [2.938–7.609]) and HFHCD mice (5.558 [4.178–7.994]); however, *p* significance in the ANOVA Kruskal–Wallis test was 0.06 (Figure 7).

## 3. Discussion

First of all, the results demonstrated that mice fed on a high-fat, high-cholesterol diet present significantly increased MMP-9 concentrations in cardiac tissue compared to mice on a standard, normal-fat diet. Such differences were not found for MMP-2, MMP-13, and MMP-14. These matrix metalloproteinases were selected due to their function: MMP-9 was chosen as it was an MMP associated with inflammation, MMP-2 and MMP-13 were chosen as they were MMPs associated with the baseline ECM protein turnover, and MMP-14 was chosen due to its role in remodeling after myocardial infarction [26,27]. Moreover, when both of the ApoE (-/-) mice groups were analyzed together, MMP-9 in cardiac tissue and MMP-9 in plasma presented a moderate and significant positive correlation, whereas MMP-9 in cardiac tissue and MMP-2 in plasma presented a slight but significant negative correlation. The dietary protocol used in our study caused significantly increased intima dimension in mice on the HFHCD compared to the NFD, which indicates that the HFHCD was efficiently proatherogenic. This proatherogenic diet only caused a significant increase in MMP-9 levels in the heart. Similarly, a study by Wang et al. demonstrated that a high-fat, high-cholesterol diet caused an increased MMP-9 expression in C57BL/6J (wild-type) mice at the level of mRNA [28]. According to the literature, MMP-9 seems to be the crucial factor in the development of cardiac fibrosis, especially after myocardial infarction or during the development of heart failure [29,30]. Moreover, MMP-9 is an important metalloproteinase involved in atherosclerotic plaque destabilization [31]. The correlation between MMP-9 cardiac and plasma levels (even though it is only significant when both the HFHCD and NFD mice were analyzed as a single group) might suggest that the intensity of processes involving this matrix metalloproteinase is also reflected in its increased plasma level. There was a study by Qin et al. that also demonstrated increased MMP-9 expression by a high-fat, high-cholesterol diet (and attenuation of this increase by simvastatin), yet it analyzed MMP-9 at the mRNA (not protein) level and did not demonstrate a correlation between cardiac and plasma MMP-9 [32]. Nevertheless, it shall be emphasized that MMP9 expression in cardiac tissue is induced by other factors (like reactive oxygen species) [33]. Interestingly, a negative, albeit slight, correlation between cardiac MMP-9 and plasma MMP-2 in the ApoE (-/-) mice on the HFHCD was also demonstrated. It shall be indicated that the role of MMP-2 is slightly different from that of MMP-9, and MMP-2 also exerts anti-inflammatory activity and a stabilizing effect on ECM turnover.

Secondly, our study demonstrated no significant differences in mRNA expression of c-Jun and TGF-β between the ApoE (-/-) mice on the NFD and HFHCD. However, significant differences in c-Jun levels were demonstrated when both the ApoE (-/-) NFD and HFHCD were compared to the C57BL/6J (WT) mice. A similar trend towards higher TGF-β expression in the WT mice (though not statistically significant), comparing both ApoE (-/-) mice groups, was also observed. A study by Yang et al. demonstrated that obesity (in the mice model obtained by a high-fat, high-cholesterol diet) increases the mRNA level of c-Jun in the blood in both humans and mice [34]. A mechanistic study by Longenecker et al. demonstrated that a mutation in TGF-β that increases its cardiac bioavailability decreases inflammation in adipose tissue and adipocyte size, although the diet itself did not alter TGF-β cardiac mRNA levels in wild-type animals [35]. The increased mRNA expression of c-Jun in the LV cardiac tissue of rats fed on a high-fat, high-cholesterol diet compared to rats fed on a normal-fat diet, with no changes in TGF-β mRNA expression between these groups, was also demonstrated by Czarzasta et al. [36]. However, in our study, the HFHCD did not cause changes in c-Jun and TGF-β expression in cardiac tissue, although a significant change was found in the MMP-9 cardiac protein level assessed by the ELISA test. These results highlight a potentially important role of MMP-9 in processes associated with the extracellular matrix within the heart, induced by a high-fat, high-cholesterol diet. However, our study demonstrated that the MMP-9 concentration in cardiac tissue is not associated with c-Jun or TGF-β expression. It might indicate that MMP-9 cardiac expression depends on other molecular pathways (such as NFκB or MAPK) or, alternatively, on the phosphorylation-mediated activation of c-Jun or TGF-β. Interestingly, our study showed that the mRNA expression of c-Jun is significantly elevated in wild-type C57BL/6 mice compared to either the ApoE (-/-) NFD or the ApoE (-/-) HFHCD. According to our knowledge, this is the first such finding and might potentially open the field for further mechanistic investigations.

## 4. Materials and Methods

### 4.1. Animals

**Figure 8:**
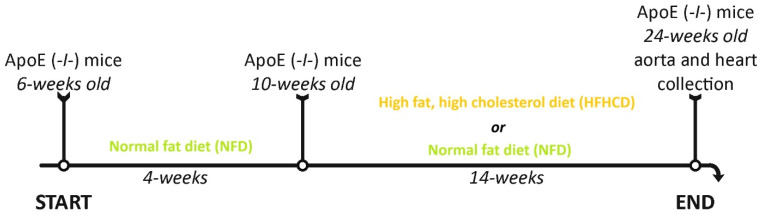
The design of the experiment showing the implementation of the high-cholesterol diet and the standard diet in the ApoE (-/-) mice.

The study was conducted on 20 male ApoE (-/-) mice (JAX^®^ Mice Strain, B6.129P2-Apoe^tm1Unc^/J, Charles Rivers Laboratories, Inc., Wilmington, MA, USA), which is a widely recognized animal model of atherosclerosis. ApoE (-/-) mice from 6 weeks of age were fed on a standard, normal-fat diet for 4 consecutive weeks. Subsequently, the mice at 10 weeks of age were divided into two groups: the control group (ApoE (-/-) NFD; *n* = 10) continued to follow the standard, normal-fat diet, while the experimental group (ApoE (-/-) HFHCD; *n* = 10) was fed on a high-fat, high-cholesterol diet (C 1090-45) for the next 14 weeks. At 24 weeks of age, ApoE (-/-) mice from both groups were euthanized to collect the abdominal aorta and heart for further analysis (Figure 8).

In addition, ten 24-week-old wild-type (WT) C57BL/6J (Clzd) mice on a standard, normal-fat diet were included in the study (WT group, *n* = 10), sacrificed, and investigated for the expression of c-Jun and TGF-β.

All of the mice were housed in individually ventilated cages (5 animals per cage; IVC, Green Line Plus; Tecniplast, Buguggiate, Italy), connected to a Smart Flow ventilation system (Tecniplast, Buguggiate, Italy) that provided 75 air changes per hour inside each cage, at a temperature of 20–24 °C, humidity 55% (±10%), and a 12 h light-dark cycle. The research study was approved by the Ministry of the Environment (Decision Number: 46/2017) for the contained use of GMO organisms, including the ApoE (-/-) mice, and approval from the First Local Ethical Committee in Warsaw, no. 50/2015. The study on animals was carried out in compliance with the ARRIVE guidelines. The animal group size was determined empirically, and the animals were randomized within each cage, with all animals in a given cage receiving the same diet. Investigators were not blinded to group allocation. The study did not establish any criteria for including and excluding animals (or experimental units) during the experiment. Therefore, all blood samples and tissue fragments obtained from the mice were submitted for further biochemical studies.

### 4.2. Feeding

The ApoE NFD mice and WT mice were fed on a standard, normal-fat diet (5% fat, 19.5% casein; metabolized energy: 3506 kcal/kg; control diet for rats and mice: C1000; Altromin Spezialfutter GmbH & Co. KG, Lage, Germany), and the ApoE HFHCD mice were fed on a high-fat, high-cholesterol diet (21% fat, 0.15% cholesterol, 19.5% casein; metabolized energy: 4432 kcal/kg; Western-Type Diet: C 1090-45; Altromin Spezialfutter GmbH & Co. KG, Lage, Germany).

### 4.3. Collection of the Abdominal Aorta and Heart

At 24 weeks of age, all of the mice were euthanized by intraperitoneal administration of ketamine (10 mg/100 g bw)/xylazine (1 mg/100 g bw). The entire aorta was isolated and fixed on 10% buffered formalin for histopathological analysis to evaluate the atherosclerotic plaque. The heart was then isolated whole, weighed, and frozen in liquid nitrogen, and then stored at −80 °C for ELISA analysis and qRT-PCR.

### 4.4. Histopathological Analysis of Atherosclerotic Plaques

**Figure 9:**
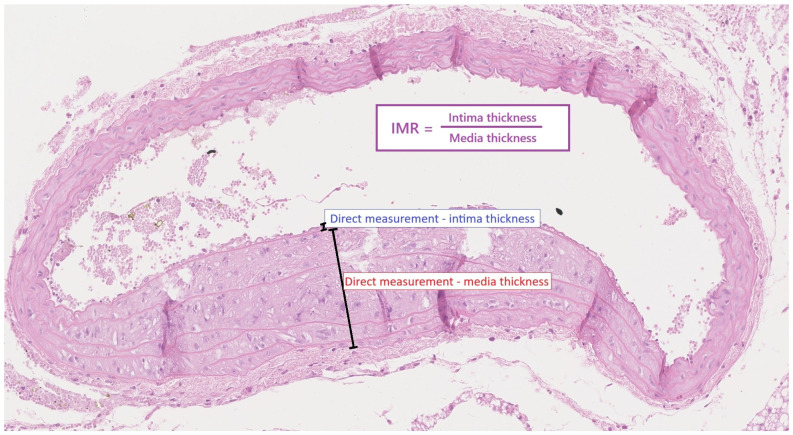
The measurement of intima thickness, media thickness, and the calculation of IMR in the aortic wall preparations of the ApoE (-/-) mice from both the NFD and HFHCD groups.

Aortic samples were fixed in 10% buffered neutral formalin (24–48 h), routinely embedded in paraffin, cut into 3 µm sections, and stained with hematoxylin and eosin (H&E) for morphological examination. The slides were digitized with a Hamamatsu NanoZooner 2.0-HT scanner (Hamamatsu Photonics, Hamamatsu, Japan) and subjectively evaluated at 5–40× magnification using NDP.view2 software, Version 2.9.29. The intima and media dimensions of the aortic wall were assessed and compared. First, the thickness of the entire circumference of the aortic intima and media was assessed. Then the measurement was performed in the thickest region, and the value of the measurement was expressed in micrometers. The intima–media ratio was calculated mathematically (Figure 9).

Immunohistochemical studies were performed using formalin-fixed, paraffin-embedded (FFPE) 3.5 μm tissue sections, and a ready-to-use antibody from Dako (Glostrup, Denmark) against smooth muscle actin (SMA, 1A4) was used. Deparaffinization, rehydration, and epitope retrieval of FFPE sections were performed in the PT Link (Dako, Glostrup, Denmark) using a pH 9 retrieval buffer. Slides were then processed with an Autostainer (Link 48 Dako, Glostrup, Denmark) using automated staining protocols validated for the antibody. Stained slides were dehydrated and automatically mounted. Special stain Masson’s trichrome was done on FFPE sections and performed using an Artisan Link Pro Special Staining System (Dako, Glostrup, Denmark) according to the manufacturer’s protocols.

### 4.5. ELISA Analysis

The MMP level in the myocardium and in plasma in both groups of ApoE (-/-) mice were determined by enzyme-linked immunoassays (ELISA), using commercially available ELISA kits: metalloproteinase-2 (MMP-2; Mouse Matrix metalloproteinase 2 ELISA Kit; MBS722437; MyBioSource, Inc., San Diego, CA, USA), metalloproteinase-9 (MMP-9; Mouse Total MMP-9 Quantikine ELISA Kit; MMPT90; R&D Systems, Inc., Minneapolis, MN, USA), metalloproteinase-13 (MMP-13; Mouse Matrix metalloproteinase 13 ELISA Kit; MBS720413; MyBioSource, Inc., San Diego, CA, USA), and metalloproteinase-14 (MMP-14; Mouse MMP-14 ELISA Kit; MBS2516088; MyBioSource, Inc. San Diego, CA, USA).

### 4.6. RT-PCR Analysis

RT-PCR analysis was conducted in accordance with the protocol previously described (Czarzasta et al.) [36]. Fragments of the heart were homogenized in TRIzol^®^ Reagent (Ambion, Life Technologies, Carlsbad, CA, USA), and the total RNA was extracted using a PureLink RNA Mini Kit (Ambion, Life Technologies, Carlsbad, CA, USA). The RNA concentration was then estimated using a Smart Spec™ Plus spectrophotometer (Bio-Rad, Hercules, CA, USA). Multiplex RT-PCR reactions were performed using the TaqMan^®^ RNA-to-CtTM1-Step Kit, a primer for the target gene (mice c jun, accession number Rn99999045_s1, Applied Biosystems, Carlsbad, CA, USA) labeled by a FAM reporter dye, and a primer for the housekeeping gene (mice GAPDH, accession number Rn01775763_g1, Applied Biosystems, Carlsbad, CA, USA) labeled by a VIC reporter dye. MicroAmp™ Optical 96-Well Reaction Plates with Barcode (Applied Biosystems, Carlsbad, CA, USA) were used. The total reaction volume was 10 μL. Amplification was conducted in 40 cycles at 95 °C for 15 s and at 60 °C for 1 min in a ViiA™ 7 Real-Time PCR System thermocycler (Applied Biosystems, Carlsbad, CA, USA). Relative gene expression was calculated from Double Delta Ct (ΔΔCt) values by relative quantification to the endogenous control.

### 4.7. Statistical Analysis

The normally distributed continuous variables were compared with Student’s *t*-test or ANOVA, the Mann–Whitney U test or the Kruskal–Wallis test was used. Bonferroni’s correction for multiple comparisons was applicable. Correlation analysis between plasma metalloproteinase concentrations and their level in the myocardium was performed using Spearman’s ρ correlation coefficient, because the variables had a distribution other than a normal distribution. For normally distributed variables, the mean and standard deviation (SD) were given. The median as well as the 25th and 75th percentiles (Q1 and Q3) were calculated. The significance level was set at 0.05. The analysis was performed using the statistical software STATISTICA 13.3 (StatSoft Inc., Tulsa, OK, USA)

## 5. Conclusions

In the present study, we showed that a high-fat, high-cholesterol diet increases MMP-9 protein concentration within the cardiac tissue in the ApoE (-/-) mice model. Moreover, MMP-9 cardiac level moderately correlates with the MMP-9 plasma level, which might indicate that increased MMP-9 cardiac expression is also at least partially reflected in plasma. The high-fat, high-cholesterol diet did not affect the cardiac c-Jun or TGF-β expression at the mRNA level; however, the mRNA level of c-Jun was significantly elevated in the wild-type C57BL/6 mice compared to their ApoE (-/-) transgenic littermates.

Work limitation: First of all, only the ApoE (-/-) mice were included in the ELISA studies (WT mice were not included); the WT mice served only as a control group for qRT-PCR analysis. Second, there were no Western blot analyses of the cardiac tissue levels of proteins and their activated, phosphorylated forms. Third, there was no analysis of mitochondrial stress in this model. Last but not least, no functional analysis of the heart (e.g., ECG) was undertaken.

## Data Availability

No new data were created or analyzed in this study. Data sharing is not applicable to this article.
